# Supplementation of vitamin E or a botanical extract as antioxidants to improve growth performance and health of growing pigs housed under thermoneutral or heat-stressed conditions

**DOI:** 10.1186/s40104-023-00981-7

**Published:** 2024-02-19

**Authors:** Ysenia V. Silva-Guillen, Consuelo Arellano, Jeffrey Wiegert, R. Dean Boyd, Gabriela E. Martínez, Eric van Heugten

**Affiliations:** 1 Department of Animal Science, North Carolina State University, Raleigh, NC 27695 USA; 2 Department of Statistics, North Carolina State University, Raleigh, NC 27695 USA; 3 Department of Animal Science, Texas A&M University, College Station, TX 77843 USA; 4 Animal Nutrition Research, LLC, Alvaton, KY 42122 USA

**Keywords:** Botanical extract, Growing pigs, Heat stress, Immune status, Oxidative status, Tocopherol, Water

## Abstract

**Background:**

Heat stress has severe negative consequences on performance and health of pigs, leading to significant economic losses. The objective of this study was to investigate the effects of supplemental vitamin E and a botanical extract in feed or drinking water on growth performance, intestinal health, and oxidative and immune status in growing pigs housed under heat stress conditions.

**Methods:**

Duplicate experiments were conducted, each using 64 crossbred pigs with an initial body weight of 50.7 ± 3.8 and 43.9 ± 3.6 kg and age of 13-week and 12-week, respectively. Pigs (*n* = 128) were housed individually and assigned within weight blocks and sex to a 2 × 4 factorial arrangement consisting of 2 environments (thermo-neutral (21.2 °C) or heat-stressed (30.9 °C)) and 4 supplementation treatments (control diet; control + 100 IU/L of D-α-tocopherol in water; control + 200 IU/kg of DL-α-tocopheryl-acetate in feed; or control + 400 mg/kg of a botanical extract in feed).

**Results:**

Heat stress for 28 d reduced (*P* ≤ 0.001) final body weight, average daily gain, and average daily feed intake (−7.4 kg, −26.7%, and −25.4%, respectively) but no effects of supplementation were detected (*P* > 0.05). Serum vitamin E increased (*P* < 0.001) with vitamin E supplementation in water and in feed (1.64 vs. 3.59 and 1.64 vs. 3.24), but not for the botanical extract (1.64 vs. 1.67 mg/kg) and was greater when supplemented in water vs. feed (*P* = 0.002). Liver vitamin E increased (*P* < 0.001) with vitamin E supplementations in water (3.9 vs. 31.8) and feed (3.9 vs. 18.0), but not with the botanical extract (3.9 vs. 4.9 mg/kg). Serum malondialdehyde was reduced with heat stress on d 2, but increased on d 28 (interaction, *P* < 0.001), and was greater (*P* < 0.05) for antioxidant supplementation compared to control. Cellular proliferation was reduced (*P* = 0.037) in the jejunum under heat stress, but increased in the ileum when vitamin E was supplemented in feed and water under heat stress (interaction, *P* = 0.04). Tumor necrosis factor-α in jejunum and ileum mucosa decreased by heat stress (*P* < 0.05) and was reduced by vitamin E supplementations under heat stress (interaction, *P* < 0.001).

**Conclusions:**

The addition of the antioxidants in feed or in drinking water did not alleviate the negative impact of heat stress on feed intake and growth rate of growing pigs.

## Background

High environmental temperatures negatively affect pig production performance, causing significant economic losses [1, 2]. During high-temperature conditions, pigs struggle to regulate core temperature because of their limited number of functional sweat glands. This condition causes pigs to be more susceptible to heat stress, which negatively affects growth performance and feed intake [3–5], increases respiration rate and rectal temperature [5, 6], reduces intestinal barrier integrity [7–9], reduces meat quality [10, 11], alters metabolism [12], and causes oxidative stress [11, 13].

Oxidative stress is defined as an imbalance in favor of oxidants as compared to the antioxidant system in the body [14]. Oxidative stress is produced by reactive oxygen species (ROS) such as hydroxyl radical, peroxyl radical, hydrogen peroxide, superoxide anion radical and singlet oxygen [15]. These molecules produce oxidation in cells, causing damage to DNA, proteins, and lipids, altering the normal functioning of the cell, and producing measurable byproducts, including 8-hydroxydeoxyguanosine (8-OHdG), protein carbonyls and malondialdehyde (MDA) [16]. The first line of defense against oxidants is the endogenous antioxidant system, which includes superoxide dismutase, catalase, and glutathione peroxidase [17]. The second line of defense is antioxidants provided in the diet, such as vitamin E, vitamin C, carotenoids, polyphenols, selenium, and zinc [17].

Vitamin E is a fat-soluble vitamin and serves as a natural antioxidant in the body. The most biologically active form of vitamin E is D-α-tocopherol [15] and DL-α-tocopheryl acetate is the most common source of vitamin E used for animal diets. Vitamin E prevents lipid peroxidation by scavenging ROS and donating electrons abstracted by free radicals from biomolecules [15]. Supplementation of vitamin E together with selenium improved intestinal epithelial barriers and alleviated oxidative stress in growing pigs housed under heat stress [18]. Vitamin E increased immune responses in broilers [19], egg production in laying hens [20] and feed intake in hens housed under heat stress [21]. On the other hand, Niu and coworkers reported no effects of vitamin E supplementation on body weight (BW), average daily feed intake (ADFI), and gain:feed ratio (G:F) in broilers housed under heat stress conditions [19]. Natural vitamin E (D-α-tocopherol) supplementation in drinking water of pigs showed high absorption of vitamin E [22, 23] and may be strategically used to decrease negative effects of heat stress in pigs when feed intake is reduced.

Polyphenols are compounds found in plants and serve to protect against insects, ultraviolet light, and physical damage [24]. Polyphenols have antioxidant properties preventing damage by ROS, can activate antioxidant enzymes and inhibit oxidases. Supplementation of polyphenols in the diet decreased MDA concentrations in plasma [25] and reduced diarrhea and *E*. *coli* excretion in weaned piglets [26]. In addition to antioxidant properties, polyphenols have been suggested to increase digestive enzyme secretions, modulate the intestinal microbiota and morphology, improve immune system functioning, and provide anti-inflammatory properties [27]. The prospect of dietary polyphenol supplementation to alleviate oxidative stress associated with heat stress through their ability to donate multiple electrons and quenching free radicals is promising and requires further study in swine.

The objective of the present study was to investigate the hypothesis that supplementation of vitamin E and a botanical extract containing polyphenols in feed or drinking water could enhance growth performance, intestinal health, and oxidative and immune status in growing pigs housed under heat stress conditions.

## Materials and methods

### Animals, housing, and experimental design

The study was conducted with a total of 128 pigs in duplicate experiments. In each experiment, 64 crossbred pigs (Smithfield Premium Genetics, Roanoke Rapids, NC, USA) equally divided into 32 barrows and 32 gilts were used. Pigs had an initial body weight of 50.7 ± 3.8 and 43.9 ± 3.6 kg and age of 13 and 12 weeks for Exp. 1 and 2, respectively. Pigs were blocked by initial BW and sex and randomly assigned within blocks to a 2 × 4 factorial randomized complete design using an experimental allotment program [28]. Therefore, there were a total of 16 blocks per treatment combination (8 blocks within each duplicate study). Factors consisted of 2 types of environments (thermo-neutral and heat-stressed), and 4 supplementation treatments applied as follows: (1) control diet (25 IU/kg of DL-α-tocopheryl acetate; CON); (2) control diet + 100 IU/L of D-α-tocopherol supplemented via the drinking water (Emcelle tocopherol, Stuart Products, Bedford, TX, USA; VEW); (3) control diet + 200 IU/kg of additional DL-α-tocopheryl acetate supplemented in the feed (Rovimix, DSM Nutritional Products, Parsippany, NJ, USA; VEF); and (4) control diet + 400 mg/kg of a botanical extract containing a variety of polyphenols supplemented in the feed (Promote, AOX 50, Cargill, Minneapolis, MN, USA; POL), based on recommended inclusion rate of the manufacturer. Supplementation of vitamin E of the control treatment corresponded to current industry recommendations [29] and the supplementation of 200 IU/kg corresponded to previous experiments [30]. Water supplementation of vitamin E was set at 100 IU/L to provide approximately the same amount of vitamin E on a daily basis as the feed supplementation, assuming a predicted water intake to feed intake ratio of 2:1.

Within each experiment, pigs and treatments were allotted randomly within blocks into 2 rooms, each containing 32 pens per room. Pigs were housed individually, resulting in 16 pigs per treatment combination for the overall study. Dietary and water treatments were equally represented in each room and were randomly distributed within room to avoid potential location effects. Pens measured 0.91 m × 1.82 m and contained a stainless-steel cup waterer (AquaChief, Hog Slat, Inc., Newton Grove, NC, USA) and an individual stainless-steel feeder (Boar feeder, Hog Slat, Inc.). All pigs were provided ad libitum access to feed and drinking water. Immediately after pigs were allocated, they were provided water supplementation or dietary treatments for 7 d prior to the initiation of temperature treatments (adaptation period). Both rooms in each experiment were set at a constant temperature of 22 °C during the adaptation period and after this period was completed, the environmental treatments (thermo-neutral and heat-stressed) were implemented for the subsequent 28 d period. The heat-stressed and thermo-neutral environmental treatments were represented by one room each within each experiment, resulting in 2 replicate rooms per environmental treatment for the study. Each room was equipped with an environmental control system (GL-5124LW Grower Direct, Monitrol, Inc., Boucherville, Quebec, Canada) to mimic high temperatures and temperature fluctuations during the day as commonly experienced in the summer season and normal thermo-neutral conditions, respectively. Temperatures for the heat-stressed room were set at 28.3, 29.4, 29.4, 31.1, 32.8, 33.3, 34.4, 35.6, 34.4, 31.7, 29.4 and 29.4 °C for 2400, 0200, 0400, 0600, 0800, 1000, 1200, 1400, 1600, 1800, 2000, and 2200 h, respectively. For the thermo-neutral room, temperatures were set at 18.9, 18.9, 20.0, 20.0, 21.1, 21.1, 22.2, 22.2, 21.1, 21.1, 20.0, and 20.0 °C for 2400, 0200, 0400, 0600 0800, 1000, 1200, 1400, 1600, 1800, 2000, and 2200 h, respectively. Temperatures were recorded every 10 min using 3 data loggers (LogTag, Micro DAQ Ltd., Contoocook, NH, USA) distributed in each room at approximately the same height as the pigs.

Dietary treatment feeds were manufactured at the North Carolina State University Feed Mill Educational Unit (Raleigh, NC, USA). Diets were primarily based on corn and soybean meal and were formulated to contain 2.78 g standardized ileal digestible lysine per Mcal ME (Table 1) and met or exceeded all nutrient requirements for growing pigs as suggested by the National Research Council [31]. A basal mix containing all ingredients, except the test ingredients, was first created and divided into 4 batches. The first and second batch were used (without any supplement) as the control treatment (CON) and the treatment receiving vitamin E in the water (VEW). Vitamin E or the botanical extract were mixed with the basal diet to create the vitamin E (VEF) and the polyphenol-containing botanical extract (POL) treatments, respectively.

**Table 1:** Composition of the experimental diets (as-fed basis)^a^

Item	CON	VEW	VEF	POL
Ingredient, %				
Corn (yellow dent)	75.63	75.63	75.63	75.63
Soybean meal (47.5% CP)	19.46	19.46	19.46	19.46
Poultry fat	1.74	1.74	1.74	1.74
Monocalcium phosphate (21% P)	1.01	1.01	1.01	1.01
Limestone	1.05	1.05	1.05	1.05
Salt	0.40	0.40	0.40	0.40
L-Lysine·HCl	0.34	0.34	0.34	0.34
DL-Methionine	0.07	0.07	0.07	0.07
L-Threonine	0.11	0.11	0.11	0.11
Mineral premix^b^	0.15	0.15	0.15	0.15
Vitamin premix^c^	0.04	0.04	0.04	0.04
Vitamin E, IU/kg	-	-	200	-
Botanical extract, mg/kg	-	-	-	400
Calculated composition				
ME, Mcal/kg	3.37	3.37	3.37	3.37
Calcium	0.63	0.63	0.63	0.63
Available P	0.27	0.27	0.27	0.27
SID Lys^d^	0.92	0.92	0.92	0.92
SID Met+Cys	0.53	0.53	0.53	0.53
SID Thr	0.58	0.58	0.58	0.58
SID Trp	0.15	0.15	0.15	0.15
Analyzed composition, %^e^				
Moisture	12.15	11.95	11.98	12.07
Crude protein	15.39	15.72	15.60	16.20
Ether extract	4.25	4.21	4.35	4.37
Crude fiber	2.17	2.12	2.34	2.39
Ash	3.96	4.24	4.42	4.12
NDF	8.74	8.05	8.44	8.99
ADF	3.39	3.22	3.63	3.42
Ca	0.63	0.68	0.75	0.69
P	0.55	0.62	0.60	0.61

^a^Diets were formulated to meet or exceed NRC (2012) [31] recommendations for 41 to 75 kg pigs. Dietary treatments consisted of control diets (CON), vitamin E supplementation in water (VEW) as D-α-tocopherol (Emcelle tocopherol, Stuart Products, Bedford, TX, USA), vitamin E supplementation in feed (VEF) as DL-α-tocopheryl acetate (Rovimix, DSM, Heerlen, The Netherlands), and a botanical extract containing various polyphenols (Promote, AOX 50, Cargill/Provimi, Minneapolis, MN, USA) in feed (POL)

^b^Supplied per kg of complete diet: 33 mg/kg of manganese, 110 mg/kg of zinc, 110 mg/kg of iron, 17 mg/kg of copper, 0.30 mg/kg of iodine, and 0.30 mg/kg of selenium

^c^Supplied per kg of complete diet: 6,614 IU of vitamin A, 1,323 IU of vitamin D_3_, 27 IU of vitamin E and 2.7 mg of vitamin K, 35 μg vitamin B_12_, 6.2 mg riboflavin, 20 mg pantothenic acid, 35 mg niacin, and 0.09 mg biotin

^d^*SID* Standardized ileal digestible

^e^Analyzed by Agricultural Experiment Station Chemical Laboratories, University of Missouri, Columbia, MO, USA

To prepare the water supplementation treatment, a stock solution was prepared by adding concentrated vitamin E to water at a ratio of 0.0256:1. The vitamin E stock solution was subsequently metered into the drinking water at a rate of 1:128 vitamin E stock solution:drinking water using a water medication device (Dosatron DM11F, Hog Slat, Inc.). Treated water was supplied to randomly selected pens (within block) within each room (8 pens per room). To determine water disappearance, both rooms were equipped with 4 water meters each. Two water meters (Elster C700 digital Invision 5/8" × 3/4" bronze valve, Elster AMCO Water, Inc., Ocala, FL, USA) were located in the principal water system (one on each side of the room) to measure water consumption for treatments that were not receiving water supplementation (24 pens in each room), and 2 water meters (water meter 5/8" Arad, AradGroup, Dalia, Israel) were used to measure water intake for the water supplementation treatment (8 pens in each room).

### Growth performance and water intake

Body weight was measured on d −7 (7 d prior to the initiation of heat stress), 0, 7, 14, and 28 to calculate ADG. Daily feed intake was measured from the difference between daily feed additions and feed remaining at the end of each weekly period divided by 7 d. Gain:feed ratio (G:F) was calculated by dividing ADG by ADFI. Water intake was determined weekly by subtracting the reading on each water meter at the beginning of the period from the reading at the end of the period.

### Respiration rate and rectal temperature

Respiration rate and rectal temperature were measured on d 0 (immediately prior to the initiation of the environmental treatments) as a baseline before heat stress was initiated. Likewise, respiration rate and rectal temperature were measured on d 1, 2, 3, 4, 5, 6, 7, 14, 21, and 28 of heat stress between 1300 and 1600 h (peak of heat stress during the day). Respiration rate was determined by counting the number of flank movements during a 30-s period at rest, using a stopwatch by the same observer during all the evaluations. Rectal temperature was measured using a digital thermometer (GLA M700, GLA Agricultural Electronics, San Luis Obispo, CA, USA) after respiration measurements were completed.

### Sample collection

Blood samples from each pig were collected by venipuncture (jugular vein) using 20-gauge × 3.8 cm drawing needles (Vacuette, Greiner bio-one, Kremsmunster, Austria) on d 2 and 28 (at 1200 h), representing short-term and long-term heat stress. Blood for serum analysis was collected into 10-mL vacuum tubes (BD Vacutainer serum, Franklin Lakes, NJ, USA). Blood was centrifuged at 4,000 × *g* for 10 min at 4 °C using a refrigerated centrifuge (Centra GP8R, Thermo IEC, Waltham, MA, USA) and serum was collected. Serum was aliquoted into 3 tubes of 2 mL capacity (Biotix, Inc., Neptune Scientific, San Diego, CA, USA) and stored at −80 °C until further analysis. Blood for complete blood cell count (CBC) analysis was collected into 6-mL vacuum tubes (BD Vacutainer containing 10.8 mg K_2_EDTA) and immediately submitted to Antech Diagnostic Laboratory (Cary, NC, USA) for analysis.

At the end of each experiment (d 28), 16 pigs in each room (total of 64 pigs; 8 pigs per experimental treatment) were euthanized using a captive bolt gun, followed by exsanguination. Blood samples were collected at the time of exsanguination of euthanized pigs and the blood samples were processed as indicated previously and stored at −80 °C. The abdominal cavity was opened, and 25 cm of the proximal jejunum (anterior to the duodenal-jejunal junction) and 25 cm of distal ileum (10 cm proximal to the ileal-cecal junction) were excised. Mucosa samples from the proximal jejunum and distal ileum were scraped using a glass slide, placed into 2-mL tubes (Biotix, Inc.), snap frozen in liquid nitrogen, and subsequently stored in a −80 °C freezer until further analysis was conducted. Four cm of intact intestinal tissue from the jejunum and ileum was collected, rinsed in 0.9% saline, and fixed in 40 mL of 10% formaldehyde solution for 3 d for histological measurements. Approximately 100 g of liver from the center of the right lobule was collected and stored in a plastic bag at −20 °C for analysis of vitamin E.

### Chemical analyses

Proximate analysis of the diets was conducted by the Agricultural Experiment Station Chemical Laboratories, University of Missouri (Columbia, MO, USA) using AOAC official methods [32]. Diets were analyzed for moisture (Method 934.01), crude protein (Method 990.03), crude fat (Method 920.39 (A)), crude fiber (Method 978.10), ash (Method 942.05), neutral detergent fiber (JAOAC 56, 1352–1356, 1973), acid detergent fiber (Method 973.18 (A–D)), calcium (Method 985.01 (A, B, D)) and phosphorus (Method 966.01).

Concentrations of vitamin E (IU/kg) in feed samples and in drinking water (IU/mL) samples were analyzed by DSM Technical Marketing Analytical Services (Belvidere, NJ, US), using a high-performance liquid chromatography system with fluorescence detection following AOAC Official Method 971.30 [32] for α-tocopherol and α-tocopheryl acetate determination in foods and feeds. Vitamin E concentrations in serum samples collected on d 2 and 28 and concentrations of vitamin E in the liver were determined by the Veterinary Diagnostic Laboratory at Iowa State University (Ames, IA, USA) using high performance liquid chromatography.

### Intestinal measurements

Cross sections of 0.4 cm thick of fixed tissue samples from the jejunum and ileum were taken and 2 to 3 sections per pig were stored into cassettes submerged in 10% formalin by the North Carolina State University College of Veterinary Medicine Histopathology Laboratory (Raleigh, NC, USA) for hematoxylin and eosin (H&E) and for Ki-67 staining of slides. Each microscope slide was photographed using an AmScope FMA050 microscope (AmScope, Irvine, CA, USA) and AmScope 3.7 software to capture and analyze images at 40× magnification. Eighteen randomly positioned villi and crypts were selected to measure villus height (from top of the villus to the crypt junction), villus width (from the middle of the length of the villus), and crypt depth (from the crypt junction to the base of the crypt) based on previously described methods [33]. Villus height and crypt depth ratio was obtained by dividing the villus height by its own crypt depth.

The proliferation rate of cells in the crypts was measured by staining for protein Ki-67, a protein located in the nucleus of proliferating cells and stained with a Ki-67 antibody. Microscope slides were scanned using 100× magnification using an AmScope FMA050 microscope and AmScope software. Images of fifteen crypts per sample were captured and evaluated using the Image JS software [34]. The ratio of Ki-67 positive cells in each crypt of the jejunum and ileum tissue was calculated by dividing Ki-67 positive cells by total cells in the crypt.

### Concentration of cytokines in mucosa and serum

Tumor necrosis factor-α (TNF-α) was measured in the mucosa of the proximal jejunum and distal ileum. Samples (0.75 to 0.80 g of mucosa) were combined with 1.5 mL of phosphate buffered saline (PBS; pH = 7.4) and subsequently homogenized (Tissuemiser, Bio-Gen PRO200, PRO Scientific Inc., Oxford, CT, USA). The samples were then centrifuged at 15,000 × *g* at 4 °C for 20 min. A 1 mL sample of supernatant was obtained and stored at −80 °C until it was analyzed. Total protein was evaluated in mucosal samples prior to analysis of TNF-α using the Pierce BCA protein assay kit (Thermo Scientific, Rockford, IL, USA). A porcine TNF-α ELISA kit (Quantitine R&D Systems, Inc., Minneapolis, MN, USA) was used to analyze TNF-α. Mucosal concentrations of TNF-α were expressed in pg/mg of total protein. The intra-assay CV were 7.3% and 4.1% for mucosal ileum and jejunum samples, respectively.

Serum samples collected on d 2 and 28 were submitted to Eve Technologies Corporation (Calgary, Canada) for analysis of pro- and anti-inflammatory cytokines using the Luminex xMAP Multi-plex technique. Cytokines analyzed included interferon (IFN)-${\upgamma }$, interleukin (IL)-1${\upalpha }$, IL-1${\upbeta }$, IL-1Ra, IL-2, IL-4, IL-6, IL-8, IL-10, IL-12, IL-18, and TNF-${\upalpha }$ and were expressed in pg/mL of serum.

### Oxidative status in mucosa and serum

Analysis of MDA was conducted in the mucosa of the ileum and jejunum and in serum. Samples of mucosa (100 mg) were homogenized using 1 mL of PBS and 10 µL of butylated hydroxytoluene. Concentrations of MDA in mucosal tissues and serum samples were analyzed using the Oxiselect TBARS assay kit protocol (MDA Quantitation; Cell BioLabs, Inc., San Diego, CA, USA). Only ileum results were measured because jejunum samples were compromised during analysis at the last step of the assay. Absorbances were measured at 532 nm in a multi-detection micro-plate reader (Synerg HT, BioTek Instruments, Winooski, VT, USA). Results from MDA for ileum mucosa and serum samples were expressed in µmol/g of total protein and µmol/L, respectively. Intra-assay CV were 2.4% and 9.0%, respectively.

### Statistical analyses

Data were analyzed using the Proc MIXED procedure of SAS (v.9.4, SAS Institute. Inc., Cary, NC, USA). Individual pig was used as the experimental unit. The model included environmental treatment, antioxidant supplementation treatments, and their interaction. Block nested within environment was used as the random effect. The least significant difference method was used to determine differences between means following a significant Fisher test. Statistical significances were considered at *P* < 0.05 and tendencies at 0.05 ≤ *P* ≤ 0.10.

## Results

### Room temperature, relative humidity, and water consumption

**Fig. 1:**
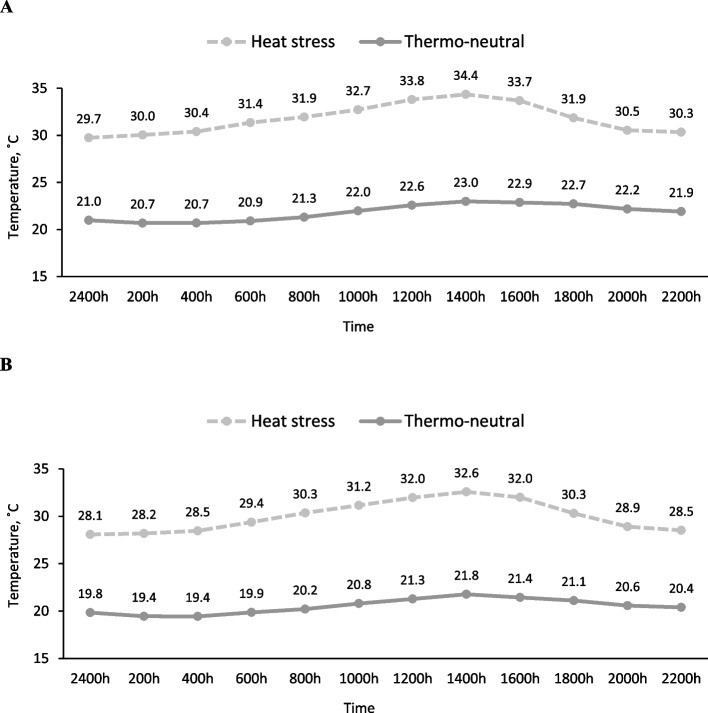
Mean temperatures of the thermo-neutral and heat-stressed environments from d 1 to 28 in Exp. 1 (panel **A**) and 2 (panel **B**). Temperatures were measured every 10 min using data recorders

The mean temperatures for the thermo-neutral room and heat stress rooms were 20.5 ± 1.66 °C and 30.0 ± 3.46 °C, respectively for Exp. 1, and 21.8 ± 3.66 °C and 31.7 ± 3.10 °C, respectively for Exp. 2. Room temperatures fluctuated within day, which was consistent with the experimental design (Fig. 1A and B). The relative humidity for the thermo-neutral room and heat stress room was 54.6% and 52.4%, respectively for Exp. 1, and 65.4% and 47.4%, respectively for Exp. 2.

Water disappearance per pig for the pigs housed in the heat stressed environment was lower than that in the thermo-neutral environment (6.7 vs. 11.3 L/d; *P* = 0.007). Water supplementation with vitamin E increased water disappearance compared to the control water when pigs were housed in the thermoneutral environment (14.3 vs. 8.4 L/d; *P* = 0.017), but water disappearance was not different due to vitamin E within the heat stressed environment (6.77 vs. 6.62 L/d).

### Growth performance

In Exp. 1, 1 pig (thermo-neutral with POL treatment) was removed from analysis due to very poor growth. In Exp. 2, 2 pigs (thermoneutral with POL and VEF treatments) were removed due to excessive weight loss related to suspected ileitis (*Lawsonia intracellularis*). Subsequently, all pigs in Exp. 2 were individually treated daily from d 9 to 21 of the experiment with an oral dose of 8.8 mg/kg BW of tiamulin hydrogen fumarate (Denagard 12.5%, Elanco Animal Health, Greenfield, IN, USA). Three pigs (2 from the thermo-neutral with VEW treatment, and 1 pig from heat-stress and control treatment), were medicated until the end of the study and 1 pig died (heat-stressed environment with VEF treatment).

Body weight (BW) was decreased (*P* ≤ 0.06) in heat stressed pigs during the last 3 weeks of the experiment, but not during the first week (Table 2). Heat stress reduced ADG and ADFI during each week (*P* < 0.04), and overall (*P* < 0.001). Gain:feed was reduced (*P* = 0.030) in pigs exposed to heat stress during the first week, but it was not impacted during the remainder of the study, or overall (*P* > 0.18). Dietary and water supplementation treatments did not significantly impact ADG, ADFI, or G:F regardless of whether pigs were housed under heat stressed or thermo-neutral conditions. In the first week of the experiment, ADG and G:F tended to increase (*P* < 0.09) by supplementation of antioxidants for pigs in the heat stress environment, but not the thermo-neutral environment (interaction, *P* = 0.051 and 0.076, respectively). In week 2, ADG and G:F for pigs provided with vitamin E in the water were lower (*P* < 0.10) compared to pigs given vitamin E or POL in the feed when pigs were housed under thermoneutral conditions, but not for heat-stressed pigs (interaction, *P* = 0.085 and 0.048).

**Table 2:** Growth performance of pigs exposed to thermo-neutral and heat-stressed environments and provided antioxidants in feed or water^a^

Item	Environment^b^								SEM	*P*-value^c^		
	Thermo-neutral				Heat-stressed							
	CON	VEW	VEF	POL	CON	VEW	VEF	POL		E	S	E × S
Body weight, kg												
d −7	46.9	47.3	47.3	47.2	47.4	47.6	47.3	47.2	0.97	0.889	0.677	0.845
d 0	52.8	54.0	52.5	52.8	53.8	54.1	53.1	53.3	1.10	0.711	0.096	0.885
d 7	60.8	61.4	59.9	60.6	58.2	59.4	58.8	58.7	1.24	0.217	0.485	0.766
d 14	68.2	67.7	67.9	68.5	64.1	65.9	64.3	64.5	1.44	0.060	0.868	0.540
d 21	75.8	76.5	75.5	76.7	69.5	71.2	70.5	70.2	1.57	0.004	0.653	0.853
d 28	82.4	83.2	81.6	83.5	74.2	76.4	75.3	75.1	1.73	0.001	0.465	0.715
Average daily gain, g/d												
d −7 to 0	843	940	735	800	916	931	816	879	60.1	0.176	0.066	0.855
d 0 to 7	1,143	1,061	1,049	1,111	631	754	820	770	54.6	<0.001	0.711	0.051
d 7 to 14	1,049	897	1,136	1,109	838	921	783	832	91.1	0.035	0.863	0.085
d 14 to 21	1,095	1,264	1,086	1,171	777	761	873	813	70.0	<0.001	0.708	0.213
d 21 to 28	935	954	875	972	672	745	690	699	57.8	<0.001	0.582	0.817
d 0 to 28	1,056	1,043	1,036	1,092	729	795	795	779	36.6	<0.001	0.554	0.370
Average daily feed intake, kg/d												
d −7 to 0	2.10	2.10	1.83	1.94	2.03	2.14	2.04	2.09	0.076	0.181	0.077	0.207
d 0 to 7	2.57	2.68	2.46	2.59	1.79	1.92	2.04	1.82	0.103	<0.001	0.663	0.206
d 7 to 14	2.71	2.61	2.71	2.85	1.94	2.08	2.01	2.02	0.129	<0.001	0.759	0.577
d 14 to 21	2.78	2.96	2.77	2.85	1.99	2.09	2.27	2.19	0.118	<0.001	0.440	0.309
d 21 to 28	2.39	2.57	2.33	2.57	1.84	1.90	1.90	1.81	0.116	<0.001	0.596	0.469
d 0 to 28	2.61	2.71	2.57	2.72	1.89	2.00	2.06	1.96	0.087	<0.001	0.584	0.408
Gain:Feed, g/kg												
d −7 to 0	393	450	399	404	448	438	397	416	24	0.388	0.302	0.533
d 0 to 7	441	396	431	431	331	385	403	418	23	0.030	0.219	0.076
d 7 to 14	368	270	423	366	438	448	367	417	48	0.189	0.707	0.048
d 14 to 21	396	439	394	417	396	358	407	384	28	0.326	0.998	0.256
d 21 to 28	398	345	378	379	363	395	365	392	25	0.793	0.927	0.396
d 0 to 28	402	389	406	405	391	401	388	405	10	0.505	0.774	0.505

^a^Values are least square means of 16 pigs. Dietary treatments consisted of control diets (CON), vitamin E supplementation in water (VEW), vitamin E supplementation in feed (VEF), and botanical extract supplementation in feed (POL). Dietary and water treatments were provided starting on d −7 and environmental treatments started on d 0

^b^Temperatures were set at the following time points: 2400, 0200, 0400, 0600, 0800, 1000, 1200, 1400, 1600, 1800, 2000, and 2200 h. Temperatures for the thermo-neutral room were 18.9, 18.9, 20.0, 20.0, 21.1, 21.1, 22.2, 22.2, 21.1, 21.1, 20.0, and 20.0 °C, and for heat-stressed room they were 28.3, 29.4, 29.4, 31.1, 32.8, 33.3, 34.4, 35.6, 34.4, 31.7, 29.4 and 29.4 °C

^c^Effect abbreviations: E = environment, S = supplementation, E × S = environment by supplementation interaction

### Respiration rate and rectal temperature

**Fig. 2:**
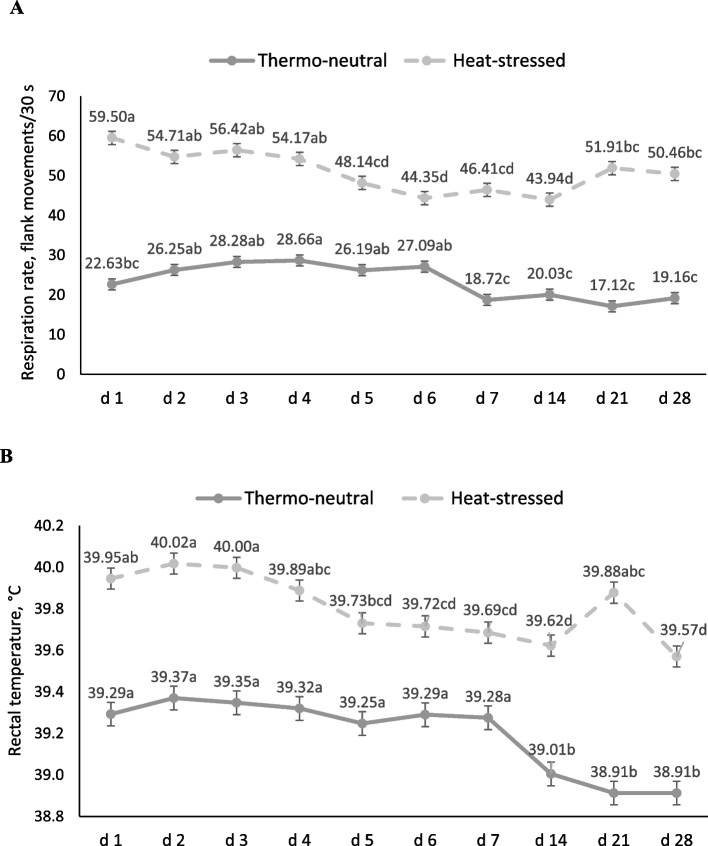
Effect of environment on respiration rate and rectal temperature measured on d 1, 2, 3, 4, 5, 6, 7, 14, 21 and 28. Environment × day interaction (*P* < 0.001). Measurements were taken between 1300 and 1600 h (peak of heat stress during the day). Numbers represent least squares means ± SEM of 64 pigs. ^a^^–^^d^Means with different superscripts are different (*P* < 0.05). **A** Respiration rate on d 0 was not different between treatments (*P* = 0.128; 19.64 and 18.36 respirations/30 s for the heat-stressed and thermo-neutral environment, respectively). Respiration rate in heat stressed pigs was greater than pigs housed under thermos-neutral conditions from d 1 through d 28. Respiration rate decreased over time within both environments. **B** Rectal temperature on d 0 did not differ between environmental treatments (*P* = 0.312; 39.26 and 39.40 ºC for the heat-stressed and thermo-neutral environment, respectively). Rectal temperatures in heat stressed pigs were greater in comparison with those in the thermo-neutral environment for all days of measurement. Rectal temperature decreased over time for both environments

Respiration rate (*P* = 0.128; 19.64 and 18.36 respirations/30 s for the heat-stressed and thermo-neutral environment, respectively) and rectal temperature (*P* = 0.312; 39.26 and 39.40 ºC for the heat-stressed and thermo-neutral environment, respectively) were not different due to environment or supplementation treatments when measured immediately prior to the initiation of heat stress. The heat-stressed environment increased (*P* < 0.001) respiration rate (Fig. 2A) and rectal temperatures in pigs (Fig. 2B). No significant differences in respiration rate or rectal temperature were detected among supplementation treatments (*P* ≥ 0.05). Respiration rate and rectal temperature decreased over the course of the experiment (*P* < 0.001) for both the thermo-neutral and heat-stressed environments, but the disparity between the thermo-neutral and heat stress environment remained throughout the study.

### Histology and immunohistochemistry in the gut

Villus height, villus width and crypt depth in the jejunum and ileum were not affected (*P* > 0.05) by environment, supplementation treatments, or their interaction (Table 3). Villus:crypt ratio in the jejunum increased by dietary vitamin E supplementation treatments compared with control (*P* = 0.046; +17.6%). Cellular proliferation measured with Ki-67 staining was greater due to heat stress in the jejunum (*P* = 0.037; +14.7%), but not in the ileum. Moreover, proliferation of enterocytes in the ileum was increased (*P* < 0.05) by dietary vitamin E and vitamin E in the drinking water for pigs housed in the heat-stressed environment compared to pigs supplemented with vitamin E in feed and water in the thermo-neutral environment (interaction, *P* = 0.043).

**Table 3:** Intestinal histology and immunohistochemistry in pigs exposed to thermo-neutral and heat-stressed environments and provided antioxidants in feed or water^a^

Item	Environment^b^								SEM	*P*-value^c^		
	Thermo-neutral				Heat-stressed							
	CON	VEW	VEF	POL	CON	VEW	VEF	POL		E	S	E × S
Villus height, μm												
Jejunum	425	381	464	402	368	359	378	394	28.9	0.119	0.368	0.514
Ileum	337	333	326	313	310	295	332	318	32.9	0.638	0.968	0.870
Villus width, μm												
Jejunum	167	155	174	168	166	153	163	156	8.3	0.391	0.314	0.882
Ileum	156	175	160	163	152	162	176	182	11.0	0.686	0.253	0.286
Crypt depth, μm												
Jejunum	148	134	138	150	130	134	106	131	11.0	0.133	0.300	0.516
Ileum	158	126	137	158	134	128	114	161	17.7	0.493	0.204	0.788
Villus:crypt ratio												
Jejunum	2.92	3.08	3.39	2.77	2.90	2.81	3.66	3.08	0.233	0.652	0.046	0.645
Ileum	2.53	3.03	2.34	2.13	2.49	2.48	3.06	2.35	0.337	0.889	0.590	0.455
Enterocyte proliferation, Ki67^d^												
Jejunum, %	43.7	38.4	41.9	41.2	48.1	47.7	48.1	45.6	2.31	0.037	0.534	0.660
Ileum, %	47.5	44.5	43.5	47.5	47.9	49.8	52.2	44.7	2.14	0.150	0.831	0.043

^a^Values are least square means of 8 pigs. Dietary treatments consisted of control diets (CON), vitamin E supplementation in water (VEW), vitamin E supplementation in feed (VEF), and botanical extract supplementation in feed (POL). Dietary and water treatments were provided starting on d −7 and environmental treatments started on d 0 until d 28

^b^Temperatures were set at the following time points: 2400, 0200, 0400, 0600, 0800, 1000, 1200, 1400, 1600, 1800, 2000, and 2200 h. Temperatures for the thermo-neutral room were 18.9, 18.9, 20.0, 20.0, 21.1, 21.1, 22.2, 22.2, 21.1, 21.1, 20.0, and 20.0 °C, and for heat-stressed room they were 28.3, 29.4, 29.4, 31.1, 32.8, 33.3, 34.4, 35.6, 34.4, 31.7, 29.4 and 29.4 °C

^c^Effects abbreviations: E = environment, S = supplementation, E × S = environment × supplementation

^d^Proliferation was evaluated by staining crypt cells with Ki67 antibody. Ki67 is a protein in the nucleus of proliferating cells

### Concentration of vitamin E in serum and liver

The concentration of vitamin E in serum was increased by supplementation (*P* < 0.001) of vitamin E in water and in feed when compared with control and the botanical extract treatments (3.59, 3.24, 1.64 and 1.67 mg/kg, respectively). Serum vitamin E concentration tended to be greater when measured on d 28 vs. d 2 (2.62 vs. 2.45 mg/kg; *P* = 0.067). When measured on d 28, vitamin E in serum was increased by supplementation of vitamin E in feed and in drinking water when compared with control and botanical extract treatment only in pigs housed in the thermo-neutral environment (interaction, *P* = 0.016), but not in the heat-stressed environment (Table 4).

**Table 4:** Concentrations of vitamin E in serum and liver and malondialdehyde (MDA) in serum and ileal mucosa of pigs exposed to thermo-neutral and heat-stressed environment and provided antioxidants in feed or water^a^

Item	Environment^b^								SEM	*P*-value^c^						
	Thermo-neutral				Heat-stressed											
	CON	VEW	VEF	POL	CON	VEW	VEF	POL		E	S	D	E×D	E×S	S × D	E × S × D
Serum vitamin E, mg/kg																
d 2	1.73	3.29	2.79	1.79	1.72	3.20	3.45	1.63	0.151	0.259	<0.001	0.067	0.016	0.584	0.001	0.038
d 28	1.64	4.01	3.63	1.77	1.49	3.86	3.11	1.48								
Liver vitamin E, mg/kg																
d 28	3.86	30.80	18.24	4.68	4.00	32.81	17.69	5.03	2.59	0.768	<0.001	NA^d^	NA	0.968	NA	NA
Serum MDA, μmol/L																
d 2	4.3	6.3	6.3	5.5	2.9	3.3	3.2	2.5	0.803	0.463	0.017	<0.001	<0.001	0.283	0.418	0.829
d 28	4.2	5.4	4.8	5.3	5.8	6.3	6.0	6.6								
Ileal mucosal MDA, μmol/g of protein																
d 28	0.362	0.208	0.185	0.159	0.180	0.183	0.225	0.229	0.04	0.568	0.156	NA	NA	0.020	NA	NA

^a^Values are least square means of 16 pigs for serum and 8 pigs for tissue measurements. Dietary treatments consisted of control diets (CON), vitamin E supplementation in water (VEW), vitamin E supplementation in feed (VEF), and botanical extract supplementation in feed (POL). Dietary and water treatments were provided starting on d −7 and environmental treatments started on d 0 until d 28

^b^Temperatures were set at the following time points: 2400, 0200, 0400, 0600, 0800, 1000, 1200, 1400, 1600, 1800, 2000, and 2200 h. Temperatures for the thermo-neutral room were 18.9, 18.9, 20.0, 20.0, 21.1, 21.1, 22.2, 22.2, 21.1, 21.1, 20.0, and 20.0 °C, and for heat-stressed room they were 28.3, 29.4, 29.4, 31.1, 32.8, 33.3, 34.4, 35.6, 34.4, 31.7, 29.4 and 29.4 °C

^c^Effects abbreviations: E = environment, S = supplementation, D = day, E × D = environment × day, E × S = environment × supplementation, S × D = supplementation × day, E × S × D = environment × supplementation × day

^d^Not applicable

Supplementation of vitamin E in feed and in drinking water increased (*P* < 0.001) the vitamin E concentration in liver tissue (Table 4). The addition of vitamin E in water increased vitamin E in the liver to a greater extent when compared to the vitamin E supplementation in the feed (*P* < 0.05). Dietary botanical extract treatment did not affect vitamin E concentration in the liver (*P* < 0.05). No significant differences (*P* ≥ 0.05) in liver vitamin E concentrations were found due to heat stress or the interaction of thermal environment and supplementation.

### Oxidative status and cytokine concentrations

The concentration of MDA in serum was increased by dietary vitamin E, vitamin E in water and dietary botanical extract treatments (*P* < 0.017), but not (*P* ≥ 0.05) by environment or the interaction between environment and supplementation (Table 4). Serum concentrations of MDA were greater (*P* < 0.001) when measured on d 28 compared with MDA levels on d 2. Moreover, heat stress reduced (*P* = 0.028) serum MDA concentrations on d 2, and, although numerically higher, it was not different (*P* = 0.213) when measured on d 28 of heat stress (interaction, *P* < 0.001). Additionally, main effects of heat stress and antioxidant supplementations were not significant for MDA concentration in ileum mucosa (*P* ≥ 0.05). However, MDA concentration in ileum mucosa was greater (*P* < 0.05) for pigs housed under thermo-neutral conditions and fed the control diet compared to all other treatments (interaction, *P* = 0.005).

Serum concentrations of IFN-γ, IL-1α, IL-1β, IL-2, IL-4, IL-6, IL-10, IL-12, IL-18, and TNF-α were not impacted (*P* ≥ 0.05) by environment, supplementation, or their interaction (Table 5). Serum concentrations of IL-8 were reduced (*P* < 0.05) and IL-1Ra concentration tended to be increased (*P* = 0.056) by heat stress, but no effects due to supplementation or interactions were observed (*P* ≥ 0.05). Serum IFN-γ and IL-8 were higher on d 28 (*P* = 0.078 and *P* < 0.001) compared to d 2. In contrast, IL-1Ra, IL-12 and IL-18 were lower (*P* < 0.05) on d 28 compared to d 2.

**Table 5:** Immune markers in serum and intestinal mucosa of pigs exposed to thermo-neutral or heat-stressed environments and provided antioxidants in feed or water^a^

Item	Day	Environment^b^								SEM	*P*–value^c^				
		Thermo-neutral				Heat-stressed									
		CON	VEW	VEF	POL	CON	VEW	VEF	POL		E	S	D	E × S	E × S × D
Serum concentration, pg/mL															
Interferon-γ	2	3,219	2,608	2,707	1,332	2,692	1,531	3,381	3,622	1003	0.375	0.587	0.078	0.306	0.383
	28	2,823	3,686	3,490	2,888	5,543	2,496	3,586	3,759						
Interleukin-1α	2	22	27	42	22	35	40	24	42	11	0.780	0.728	0.284	0.364	0.463
	28	15	25	36	34	25	29	28	21						
Interleukin-1β	2	208	267	392	200	254	252	223	456	79	0.827	0.105	0.133	0.392	0.113
	28	136	210	210	346	200	128	260	301						
Interleukin-1Ra	2	738	672	690	456	953	814	716	846	105	0.056	0.871	< 0.001	0.274	0.615
	28	345	409	571	467	495	614	419	533						
Interleukin-2	2	94	153	228	92	151	120	109	260	60	0.891	0.629	0.266	0.507	0.058
	28	54	145	102	189	131	72	159	103						
Interleukin-4	2	525	730	1,520	432	628	732	916	1,592	438	0.801	0.375	0.217	0.906	0.059
	28	237	702	461	1,127	562	325	1,152	471						
Interleukin-6	2	13	21	36	14	21	16	21	34	11	0.924	0.449	0.281	0.813	0.388
	28	5	14	28	20	11	21	24	9						
Interleukin-8	2	428	450	377	410	318	344	292	343	75	0.007	0.359	< 0.001	0.282	0.508
	28	688	574	679	652	394	415	534	738						
Interleukin-10	2	248	328	565	222	376	260	264	613	131	0.802	0.561	0.164	0.610	0.033
	28	177	270	223	408	300	169	402	240						
Interleukin-12	2	1,224	1,499	1,526	1,139	1,324	1,392	1,346	1,401	117	0.807	0.221	0.001	0.669	0.257
	28	1,135	1,153	1,285	1,183	1,063	1,274	1,161	1,057						
Interleukin-18	2	803	924	1,727	808	783	815	611	1,141	232	0.324	0.482	0.002	0.250	0.023
	28	294	741	638	927	673	456	639	510						
TNF-α^d^	2	78	57	69	78	83	46	43	59	32	0.920	0.983	0.576	0.968	0.802
	28	37	60	49	55	26	64	99	48						
Mucosal TNF-α															
Jejunum, pg/mg protein	28	847	1,094	786	517	412	1,112	191	723	197	0.022	0.064	NA^e^	0.535	NA
Ileum, pg/mg protein	28	5.6	6.4	2.3	2.4	1.5	2.1	2	2.8	0.45	0.009	< 0.001	NA	< 0.001	NA

^a^Values are least square means of 16 pigs for serum and 8 pigs for tissue measurements. Dietary treatments consisted of control diets (CON), vitamin E supplementation in water (VEW), vitamin E supplementation in feed (VEF), and botanical extract supplementation in feed (POL). Dietary and water treatments were provided starting on d −7 and environmental treatments started on d 0 until d 28

^b^Temperatures were set at the following time points: 2400, 0200, 0400, 0600, 0800, 1000, 1200, 1400, 1600, 1800, 2000, and 2200 h. Temperatures for the thermo-neutral room were 18.9, 18.9, 20.0, 20.0, 21.1, 21.1, 22.2, 22.2, 21.1, 21.1, 20.0, and 20.0 °C, and for heat-stressed room they were 28.3, 29.4, 29.4, 31.1, 32.8, 33.3, 34.4, 35.6, 34.4, 31.7, 29.4 and 29.4 °C

^c^Effects abbreviations: E = environment, S = supplementation, D = day, E × D = environment × day, E × S = environment × supplementation, S × D = supplementation × day, E × S × D = environment × supplementation × day. Effects without significant differences or tendencies were not shown

^d^Tumor necrosis factor-α

^e^Not applicable

The concentration of TNF-α in mucosa of the jejunum was decreased (*P* = 0.022) by the heat-stressed environment, and the supplementation of vitamin E in water tended to increase (*P* = 0.064) TNF-α in jejunum mucosa (Table 5). TNF-α concentration in the mucosa of the ileum was decreased by the heat-stressed environment (*P* < 0.05) and vitamin E supplementation in the water (*P* < 0.001), but not dietary vitamin E or botanical extract treatments. TNF-α was reduced in the ileum mucosa by vitamin E supplementation, but not dietary botanical extract in the heat-stressed environment (interaction, *P* < 0.001).

### Complete blood count (CBC)

Red blood cells, hemoglobin, and hematocrit percentage were reduced on d 28 by the heat-stressed environment, but this was not the case on d 2 (interaction, *P* < 0.05; Table 6). White blood cells, platelets, neutrophils, and monocytes counts were lower (*P* < 0.001) on d 28 compared to d 2, but no other differences were observed.

**Table 6:** Complete blood count measured on d 2 and d 28 in pigs exposed to thermo-neutral or heat-stressed environments and provided antioxidants in feed or water^a^

Item	Day	Environment^b^								SEM	*P*-value^c^			
		Thermo-neutral				Heat-stressed								
		CON	VEW	VEF	POL	CON	VEW	VEF	POL		E	S	D	E × D
White blood cells, 10^3^/μL	2	17.2	18.8	17.9	17.5	17.9	17.7	17.5	16.0	1.13	0.581	0.255	<0.001	0.840
	28	14.8	13.3	14.1	12.8	13.8	13.8	13.4	12.5					
Red blood cells, 10^6^/μL	2	6.65	6.83	6.71	6.51	6.84	6.76	6.73	6.58	0.22	0.287	0.750	0.430	0.020
	28	6.94	6.94	6.80	7.33	6.57	6.70	6.42	6.56					
Hemoglobin, g/dL	2	12.14	12.45	12.37	11.97	12.21	12.34	12.25	12.21	0.37	0.107	0.563	0.188	0.003
	28	12.73	12.98	12.79	13.49	11.72	12.09	11.98	11.99					
Hematocrit, %	2	40.6	41.6	41.2	39.3	40.5	40.9	41.2	40.4	1.37	0.152	0.767	0.670	0.013
	28	42.0	42.1	41.8	44.8	39.0	40.2	38.5	39.5					
Platelet Count, 10^3^/μL	2	252	260	242	247	312	270	305	282	31	0.268	0.542	<0.001	0.382
	28	219	169	180	170	205	217	219	175					
Neutrophils, count/μL	2	7,223	6,559	6,353	6,401	6,471	6,430	5,969	5,221	572	0.247	0.215	<0.001	0.658
	28	3,908	3,622	4,426	3,231	3,336	3,795	3,525	3,017					
Lymphocytes, count/μL	2	8,272	9,503	9,667	9,269	9,782	9,528	9,609	8,972	731	0.795	0.758	0.123	0.652
	28	9,786	8,540	8,462	8,396	9,164	8,850	8,676	8,415					
Monocytes, count/μL	2	1,219	1,256	1,142	1,226	1,002	1,121	1,339	1,289	118	0.647	0.889	<0.001	0.768
	28	736	720	754	733	771	651	705	590					
Eosinophils, count/μL	2	400	435	653	519	582	531	499	468	99	0.500	0.702	0.304	0.336
	28	348	412	458	422	534	535	502	497					

^a^Values are least square means of 16 pigs. Dietary treatments consisted of control diets (CON), vitamin E supplementation in water (VEW), vitamin E supplementation in feed (VEF), and botanical extract supplementation in feed (POL). Dietary and water treatments were provided starting on d −7 and environmental treatments started on d 0 until d 28

^b^Temperatures were set at the following time points: 2400, 0200, 0400, 0600, 0800, 1000, 1200, 1400, 1600, 1800, 2000, and 2200 h. Temperatures for the thermo-neutral room were 18.9, 18.9, 20.0, 20.0, 21.1, 21.1, 22.2, 22.2, 21.1, 21.1, 20.0, and 20.0 °C, and for heat-stressed room they were 28.3, 29.4, 29.4, 31.1, 32.8, 33.3, 34.4, 35.6, 34.4, 31.7, 29.4 and 29.4 °C

^c^Effects abbreviations: E = environment, S = supplementation, D = day, E × D = environment × day, E × S = environment × supplementation, S × D = supplementation × day, E × S × D = environment × supplementation × day. Interactive effects without significant differences or tendencies were not shown

## Discussion

Heat stress reduces growth performance in pigs as demonstrated in many studies [1, 2, 4, 5, 35]. In the present study, the impact of heat stress on BW could not be detected after 7 days of exposure, but clearly and consistently reduced BW of pigs when measured in subsequent weeks, ultimately resulting in a reduction of 7.4 kg (9% reduction) at the end of the 28-day study. The impact of heat stress has been reported to be dependent on pig body weight, with a greater negative impact in heavier pigs [5]. The reduction in ADFI associated with heat stress is the major contributor to decreased growth performance, although reduced ADFI does not always completely account for the decreased growth performance [2, 36]. During high temperature environments, the body reacts by decreasing or avoiding any extra heat production that could increase core body temperature, including high feed intake. In the present study, ADG and ADFI in growing pigs were decreased by 26.7% and 25.4% due to heat stress, without impacting feed efficiency. These results are consistent with other report in growing pigs [2, 3, 35–38].

Clearly heat stress reduced performance in the present study and we hypothesized that the use of antioxidants could ameliorate, in part, the negative effects of heat stress in growing pigs. However, the supplementation of vitamin E in water and the supplementation of vitamin E and botanical extract in feed did not affect BW, ADFI, or G:F, regardless of environmental temperature. Niu and co-workers reported that the addition of vitamin E in the diet did not affect BW or ADFI, but G:F was decreased using 100 mg/kg of dietary vitamin E in broilers and no effects were observed using 200 mg/kg of vitamin E, regardless of heat stress [19]. In growing pigs, dietary supplementation with vitamin E reduced feed efficiency, but no statistical differences were detected for ADG and ADFI [39]. Inclusion of dietary polyphenols (from grape pomace included at 7.5% [40] and 0.1% of a blended polyphenol additive [25]) did not show any significant differences on growth performance when used in broilers and weaned piglets, respectively. In the present study, the botanical extract containing polyphenols did not affect growth performance of pigs. The response to dietary polyphenols can be affected by differences in absorption, metabolism, and interaction with other nutrients [24]. Indeed, there are many different polyphenolic compounds with potential promising impacts on health, immune response, microbial balance, antioxidant status, and ultimately growth performance. However, these supplements need to be closely characterized in terms of concentrations of active compounds, where they were derived from and by what specific method, followed by clearly defined experimental protocols aimed at evaluating their efficacy [27].

The use of water by pigs to drink and spray themselves to reduce core body temperature is expected to be higher under a high temperature environment. Although it was not a primary objective of the present study, the estimated disappearance of drinking water for pigs housed in the heat-stressed environment was 40.7% lower than the thermo-neutral environment. Pigs in the present study only had access to cup waterers with a nipple inside the cup, specifically to minimize water wastage associated with behavioral changes such as wetting of the skin to increase evaporative heat losses. It should also be noted that the water in the heat stress rooms was warm due to the high temperature of the rooms, which may have caused the lower water consumption of heat stressed pigs compared to the pigs housed in the thermo-neutral room. Others reported decreased water consumption in pigs during hot temperatures [6, 41] and reduced water intake in pigs when the drinking water temperature was warm compared to cold water [42]. Supplemental vitamin E appeared to increase water disappearance within the thermoneutral rooms, but not within the heat stressed rooms.

High respiration rate and rectal temperature are positively correlated with heat stress in pigs when temperatures exceed 25 ºC temperature [3, 18, 43, 44]. High body temperature is associated with thermoregulatory mechanisms sending blood flow to the periphery to dissipate the excess heat [45]. In the present study, heat stress clearly increased respiration rate and rectal temperature throughout the study, but antioxidant supplementation did not ameliorate these effects. In the present study, some acclimation to the heat-stress and thermo-neutral conditions was observed as indicated by a reduction in rectal temperature and respiration rate over time, similar to other studies [43, 46].

Heat stress causes damage in the intestine due to a redistribution of blood flow to the periphery to dissipate heat, reducing blood flow to the splanchnic organs. Heat stress has been shown to cause damage to the tips of jejunal villi, shortening of villus height and decreasing crypt depth [35, 44], damaging duodenal epithelium [7], and compromising intestinal integrity [9]. The impact of heat stress on intestinal dysfunction has recently been confirmed and it was further demonstrated that these impacts were closely related to alterations in intestinal microbiota [41]. Contrarily to these reports, no effects of heat stress on histology in the ileum or jejunum were detected in the current study. However, heat stress increased cell proliferation in the jejunum as measured by Ki-67 staining. The addition of dietary vitamin E increased the villus:crypt ratio in the jejunum, but dietary supplementation with the botanical extract did not alter intestinal histology. Gessner and coworkers showed significant increases in villus height:crypt depth ratio in the duodenum of 6-week-old piglets when using polyphenols (10 g/kg of grape seed and grape marc extract) in the diet [47]. The addition of vitamin E in feed and in water improved cell proliferation in the ileum of pigs housed under heat stress condition, but not in pigs housed under thermo-neutral conditions, suggesting that the body accelerated cellular proliferation to compensate for cellular death by hypoxia during heat stress when vitamin E, but not the botanical extract, was supplemented.

Several authors reported that heat stress reduced serum vitamin E concentration [20, 48], presumably because vitamin E reacts against oxidation caused by heat stress, reducing its concentration in serum. In addition, reduced vitamin E intake due to an overall decrease in feed consumption associated with heat stress is expected to have a significant impact on vitamin E status. In the current study, serum vitamin E concentration was reduced from 2.76 to 2.48 mg/kg due to prolonged heat stress when measured on d 28, but not during short-term heat stress on d 2. Similarly, vitamin E concentrations were greater on d 28 compared to d 2 in pigs housed under thermoneutral conditions, but not in heat-stressed pigs. Liver concentrations of vitamin E were not impacted by heat stress, in spite of the significant impact of heat stress on pig performance, including a substantial reduction in feed intake and thus, reduced vitamin E intake. Serum and liver vitamin E concentrations were increased with vitamin E supplementation, especially when vitamin E was supplemented in the drinking water compared to dietary vitamin E. The high concentrations of vitamin E in serum and liver with vitamin E supplementation in water could be due in part to the fact that the natural form of vitamin E (D-α-tocopherol) that was used is more bioavailable than the synthetic form (DL-α-tocopheryl acetate) that was used in the feed. Similarly, Wilburn et al. reported greater concentrations of vitamin E in serum and liver when using natural RRR-α-tocopheryl acetate in water compared to the synthetic all-*rac*-α-tocopheryl acetate [23], confirming very efficient absorption of vitamin E when it is supplemented in the water [22, 49]. In addition, total intake of vitamin E per day, using the estimated water consumption of pigs supplemented with vitamin E in the water was 900 IU compared to 500 IU total vitamin E intake when supplemented in feed. Thus, part of the response is likely related to greater vitamin E intake when it was supplemented in the water. The addition of the botanical extract did not impact serum vitamin E concentrations similar to other reports [49, 50], suggesting that the botanical extract used in the current study was not effective in sparing or regenerating vitamin E. On the other hand, Luehring et al. [51] showed that polyphenols in combination with low dietary vitamin E increased vitamin E in plasma and in liver of growing pigs when using fish oil to induce oxidative stress. Lack of response to polyphenols could be related to low absorption rate of dietary polyphenols [44], the type of and activity of polyphenols used [27], or antioxidant functioning of polyphenols independent from vitamin E.

Malondialdehyde is a product produced during lipid peroxidation in the cell under oxidative stress [52]. In a study conducted by Montilla et al. [10], MDA was 2.5-fold greater in grower pigs (35 kg body weight) during a short 1-day period of heat stress compared with a thermo-neutral environment. In the present study, heat stress reduced serum MDA concentrations after short-term exposure, but not after longer-term heat stress. The reduction of MDA concentrations during short-term heat stress suggested that the enzymatic (superoxide dismutase, catalase, glutathione peroxidase) and nonenzymatic (vitamin A and vitamin E) antioxidant systems reacted effectively against oxidation, but that this could not be fully maintained during prolonged heat stress [17]. The inclusion of other dietary antioxidants, such as polyphenols, reduced MDA levels in broilers and piglets in muscle, liver, and plasma [51, 53, 54]. In contrast, supplementation with the botanical extract or vitamin E either in feed or drinking water increased serum MDA concentrations when compared to the other treatment. Other studies found that vitamin E and polyphenol-based antioxidants did not affect MDA concentrations in loin muscle in finishing pigs and diabetic or not diabetic rats, and in piglets [39, 49, 55, 56].

In the present study, no effects on MDA concentrations in the ileum due to heat stress or supplementation were observed. Lambert et al. reported no increase in lipid peroxidation products in the small intestine of rats housed under high temperatures (42.5 ºC) [57]. Contrarily, Maini and co-workers [58] found that adding 200 IU dietary vitamin E to diets fed to broilers under heat stress reduced MDA concentrations due to amelioration of enzymatic and nonenzymatic antioxidant system by the vitamin E. On the other hand, Ebrahimzadeh and others [40] showed a greater reduction in MDA levels when using polyphenols (7.5% of grape pomace) than vitamin E (200 mg/kg of α-tocopherol acetate feed) in broilers. Intestinal cells pre-treated in vitro with Trolox (a water-soluble analogue of vitamin E) showed markedly reduced oxidative stress when compared with intestinal cells pre-treated in vitro with ascorbic acid [58].

Tight junctions provide structural integrity and barrier function in the intestinal epithelium. When they are dysregulated by heat stress, it causes alterations in the barrier function, producing pro-inflammatory and anti-inflammatory cytokines [59]. Thus, under heat stress, the pro-inflammatory cytokine TNF-α is produced [60]. In the present study, TNF-α in the ileum and jejunum was reduced by the heat-stress environment. We can speculate that the reduction of TNF-α in ileum and jejunum under heat stress can be due to the inhibition of NF-κB (nuclear factor kappa-light-chain) or that the peak of TNF-α occurred before the tissues were collected on d 28. Bouchama et al. [60] and Liu et al. [18] did not find significant changes in TNF-α in jejunum and ileum of pigs housed under heat stress when using dietary vitamin E and selenium. In the present study, supplementation of vitamin E in feed and in water resulted in a reduction of TNF-α when compared to the rest of the dietary treatments for pigs housed under heat stress. This may suggest that dietary supplementation with vitamin E reduced some inflammation in tissue of pigs during heat stress.

Serum TNF-α, IL-1α, IL-1β, IL-2, IL-4, IL-6, and IL-10 were not affected by heat stress, dietary supplementation, or day of measurement. Perhaps, the heat-stressed environment in the present study was not severe enough to produce inflammation in the body that could be detected in serum. Additionally, our previous work [49] did not find effects on serum TNF-α in weaned piglets supplemented with dietary vitamin E and polyphenols. In contrast, Gabler et al. [61] reported significantly lower serum TNF-α levels in pigs housed under heat stress on d 3. Likewise, Pearce et al. [62] showed a reduction in TNF-α in serum of growing pigs under heat stress due to the inhibition of NF-κB enhance of activated B cell by heat shock proteins produced by heat stress. Heat exposure reduced TNF-α in the ileum suggesting that heat stress had effects at the local tissue level and probably could not be detected in serum. Thus, the expression of mucosal TNF-α can be different than circulating TNF-α [9, 63]. Similarly, TNF-α concentrations in serum were not affected by heat exposure for increasing duration (0, 2, 4 and 6 h) in finishing pigs [35].

In the present study, serum IFN-γ concentration increased on d 28 but IL-12 and IL-18 were reduced on d 28 compared to d 2, showing low inflammatory responses, even though IL-12 and IL-18 act synergistically inducing IFN-γ [64]. Additionally, IL-8, a pro-inflammatory cytokine and activator of neutrophils in local inflammation [65] was reduced in serum by heat stress and increased on d 28 compared to d 2. In contrast, Liu et al. [18] did not observe changes in IL-8 in the jejunum and ileum of 20-kg pigs when exposed to 20 ºC or 35 ºC using dietary vitamin E and selenium. IL-1Ra is a natural anti-inflammatory cytokine protein which increases during inflammation [66] and has an antagonist effect on IL-1β and IL-1α [64]. In the present study, serum IL-1Ra increased due to heat exposure, and IL-1Ra was reduced on d 28 compared to d 2. Based on this result, heat exposure produced some inflammation to increase IL-1Ra in serum to counteract this inflammation in the pigs. Also, the reduction of IL-1Ra on d 28 suggests the early potential presence of injurious components in the body [67] with the following resolution by d 28.

Red blood cell count, hemoglobin and hematocrit percentage rise or fall altogether, and increase due to deprivation of drinking water or decrease due to blood loss [68]. Red blood cells have high polyunsaturated fatty acids in their membranes and can be affected by oxidative stress and serving their high concentrations of oxygen as ROS precursors [69]. In the present study, red blood cell count, hemoglobin and hematocrit percentage were reduced by 1.5%, 3.0% and 3.7%, respectively by the heat stress environment on d 28. Likewise, Mendoza et al. [3] observed a small reduction of 1% in red blood cells, hemoglobin and hematocrit due to heat stress in 39-kg BW pigs. Also, Adenkola et al. [70] showed a reduction of 19% in red blood cells during thermally stressful environmental conditions in adult pigs by 3 months (harmattan season). Thus, in the present study, the reduction of red blood cell count, hemoglobin and hematocrit in the heat-stressed environment at d 28 could be associated with the oxidation of polyunsaturated fatty acids in the red blood cells by heat stress [69] and impaired synthesis of hemoglobin [71]. Even though the heat stressed pigs had reduced ingestion of water and possibly dehydration, this fact was not significant enough to elevate red blood cell count, hemoglobin and hematocrit. All CBC values were within normal ranges [72]. Platelets are involved in aggregation and clot formation and immunity [68, 71]. Habibu et al. [71] reported a reduction in platelet count due to heat stress in cattle and ducks. In the present study, platelets were not impacted by heat stress, but they were reduced by 40% on d 28 compared to d 2 with the total values being 29% below the normal range [72].

White blood cells play a critical role in the immune system. In the present study, white blood cells, neutrophils, and monocytes were not affected by heat stress, but they were decreased on d 28 by 29%, 75%, and 69%, respectively, compared to d 2. In contrast, Mendoza et al. [3] reported reductions in neutrophils (−10%) due to heat stress. Adenkola et al. [70] reported increased numbers of white blood cells, neutrophils, but no differences in monocytes, during the hot-dry season (temperatures between 30–34 ºC) in adult pigs. In the present study, the reduction in white blood cells, neutrophils, and monocytes on d 28 can be due to a resolution of a potential injury in the pigs, even though values were within normal ranges [72].

In this study, the supplementation of vitamin E in feed and in water, and a dietary botanical extract containing a variety of polyphenols did not affect red blood cells, hemoglobin, hematocrit, white blood cells, neutrophils, monocytes, or platelets. Attia et al. [73] did not find significant differences in complete blood count when dietary vitamin E was supplemented in the feed of broilers under heat stress. Likewise, Stukelj et al. [74] did not observe changes in hematological parameters of 7-week pigs when dietary polyphenols were supplemented in the diet.

## Conclusions

Heat stress clearly increased rectal temperature and respiration rate, which persisted throughout the study, and decreased growth performance of pigs resulting in reduction in body weight of 7.4 kg during the 28-day study. The negative impact of heat stress on growth rate was primarily related to a reduction in feed consumption. In spite of the significant negative impact of heat stress on growth performance, there were no clear or consistent effects of heat stress on oxidative stress, serum cytokines, or intestinal morphology. Supplementation of vitamin E increased serum and liver concentrations of vitamin E, especially when provided via the water, but the polyphenol-containing botanical extract was not effective in improving vitamin E status. However, nutritional supplementation was not effective in improving growth performance, oxidative stress, or immune markers. Heat stress showed limited impacts on oxidative stress, intestinal morphology, and immune markers, which may have limited the potential impact of nutritional supplementation with vitamin E and plant-based polyphenols from the botanical extract. The addition of the antioxidants in feed or in drinking water in the current study did not ameliorate the negative effects caused by heat stress in growing pigs.

## Data Availability

The datasets analyzed in the present study can be made available from the corresponding author upon reasonable request.

## Abbreviations

ADFI: Average daily feed intake
ADG: Average daily gain
BHT: Butylated hydroxytoluene
BW: Body weight
CBC: Complete blood count
DNA: Deoxyribonucleic acid
G:F: Gain:feed ratio
IFN: Interferon
IL: Interleukin
MDA: Malondialdehyde
ROS: Reactive oxygen species
SID: Standardized ileal digestible
TNF-α: Tumor necrosis factor-α
